# *In vitro* activity of gepotidacin against urinary tract infection isolates of Enterobacterales, *Enterococcus faecalis*, and *Staphylococcus saprophyticus*

**DOI:** 10.1128/aac.00296-25

**Published:** 2025-05-15

**Authors:** Meredith A. Hackel, James A. Karlowsky, Daniel F. Sahm, Joshua M. West, Nicole E. Scangarella-Oman

**Affiliations:** 1 IHMA 356047 , Schaumburg, Illinois, USA; 2 Department of Medical Microbiology and Infectious Diseases, Max Rady College of Medicine, University of Manitoba 574854, Winnipeg, Manitoba, Canada; 3 GSK 73111 , Collegeville, Pennsylvania, USA; The Peter Doherty Institute for Infection and Immunity, Melbourne, Victoria, Australia

**Keywords:** gepotidacin, triazaacenaphthylene, type II topoisomerase inhibitor, urinary tract infections

## Abstract

Gepotidacin is a novel, bactericidal, first-in-class triazaacenaphthylene antibiotic that inhibits bacterial DNA replication through a distinct binding site and unique mechanism of action, providing well-balanced inhibition of two different type II topoisomerase enzymes for most uropathogens. Phase III clinical trials, NCT04020341 (EAGLE-2) and NCT04187144 (EAGLE-3), showed gepotidacin to be non-inferior and superior, respectively, to nitrofurantoin for the treatment of patients with uncomplicated urinary tract infections (uUTIs). To better define gepotidacin *in vitro* activity against pathogens that commonly cause UTIs, CLSI broth microdilution MICs were determined for gepotidacin and seven comparator agents, and agar dilution MICs were determined for fosfomycin, against 4,000 predominantly UTI isolates of Enterobacterales (3,250), *Enterococcus faecalis* (500), and *Staphylococcus saprophyticus* (250) collected globally from 2012 to 2020. Gepotidacin MIC_90_s against the Enterobacterales species tested were 4 µg/mL for *Escherichia coli* (1,000) and *Klebsiella oxytoca* (250), 8 µg/mL for *Citrobacter* spp. (250) and *Klebsiella aerogenes* (250), 16 µg/mL for *Proteus mirabilis* (250) and *Providencia rettgeri* (250), and 32 µg/mL for *Enterobacter cloacae* (500) and *Klebsiella pneumoniae* (500). Against the gram-positive species, gepotidacin MIC_90_s were 0.12 µg/mL and 4 µg/mL for *S. saprophyticus* (250) and *E. faecalis* (500), respectively. Gepotidacin MIC_90_s for ciprofloxacin not susceptible isolates ranged from 4 µg/mL for *E. coli* (352) to 128 µg/mL for *P. rettgeri* (48). Gepotidacin MIC_90_s for presumptive extended spectrum beta-laactamase (ESBL)-positive *E. coli* (228) and *K. pneumoniae* (145) were 8 µg/mL and 32 µg/mL, respectively. Gepotidacin was bactericidal (minimum bactericidal concentration [MBC]/MIC ratio ≤4) against 94% (47/50) of isolates tested.

## INTRODUCTION

Urinary tract infections (UTIs) are common in reproductive age and postmenopausal women (1, 2). Uncomplicated UTIs (uUTIs) are defined as infections arising in otherwise healthy, non-pregnant, pre-menopausal, ambulatory adult women with no history suggestive of functional or anatomical urinary tract abnormality (1). Approximately 11% of women >18 years of age experience at least one episode of uUTI per year, with the peak incidence of infection in young, sexually active women (3). An estimated one-third of all female adolescents and women experience at least one episode of uUTI requiring antibiotics by the age of 24 years (4, 5). The morbidity associated with UTI, particularly for the 20%–30% of individuals experiencing recurrent infection, is significant and affects patient quality of life (1, 2). The predominant uropathogens isolated from urine collections of patients with community-acquired uncomplicated cystitis and pyelonephritis are *Escherichia coli* (75%–90%) and *Staphylococcus saprophyticus* (5%–15%); *Enterococcus faecalis*, *Klebsiella pneumoniae*, and other species of Enterobacterales are identified less frequently (1, 5).

Initial therapy for uUTI is based on multiple factors including severity of illness, prior treatment history, previous microbiology results, and local or national antibiogram data. Currently recommended first-line empiric therapies to treat uUTIs are trimethoprim-sulfamethoxazole, nitrofurantoin, and fosfomycin; second-line therapies include β-lactams and fluoroquinolones (1). However, these guidelines were published more than 10 years ago (in 2011). Since then, resistance rates for trimethoprim-sulfamethoxazole and fluoroquinolones in *E. coli* have increased to >20%, and ESBL rates in both *E. coli* and *K. pneumoniae* are approaching 10% (ESBLs confer resistance to both oral and parenteral cephalosporins as well as piperacillin-tazobactam), which may preclude these agents from consideration for empiric therapy (2). This, together with the limited spectra of activity of nitrofurantoin and fosfomycin (6), and the increase in multidrug-resistant isolates, underlies the need for the development of new antibacterial agents, particularly agents with novel mechanisms of action and which are ideally deliverable in an oral formulation (7, 8).

Gepotidacin is the first antibacterial in a new class known as triazaacenaphthylenes and has a homologous binding site in both bacterial DNA gyrase (an enzyme that creates negative supercoils in DNA mediated by the C-terminal domain of its DNA binding subunit [GyrA]) and topoisomerase IV (an enzyme that decatenates DNA and relaxes positive supercoils mediated by its ParC subunit) that is distinct from that of the fluoroquinolones (9, 10). Additionally, the gepotidacin mechanism of action is unique from any currently approved agent, including fluoroquinolones (9, 10). For most uropathogens, gepotidacin demonstrates well-balanced inhibition of two type II topoisomerase enzymes (DNA gyrase and topoisomerase IV) (11, 12). A distinct gepotidacin binding site and unique mechanism of action confer activity against most target pathogens resistant to established antimicrobial agents, including fluoroquinolones (13–18).

Gepotidacin has completed Phase III clinical trials (NCT04020341 [EAGLE-2] and NCT04187144 [EAGLE-3]) as an oral treatment for uUTI and was shown to be non-inferior to nitrofurantoin in NCT04020341 and superior to nitrofurantoin in NCT04187144 (19). Gepotidacin also recently completed a Phase III clinical trial as an oral treatment for uncomplicated urogenital gonorrhea (NCT04010539). To better define gepotidacin *in vitro* activity against a broad collection of pathogens (collected globally from 2012 to 2020) commonly associated with uUTIs, we determined MICs for gepotidacin and eight comparator agents against 3,250 Enterobacterales, 500 *E. faecalis*, and 250 *S*. *saprophyticus* isolated primarily from patients with UTI. In addition, minimum bactericidal concentration (MBC) determinations were performed on a randomly selected subset of 50 isolates from the larger collection.

## RESULTS

MIC values for gepotidacin against all 3,250 Enterobacterales isolates tested ranged from 0.06 to >128 µg/mL (Tables 1 and 2). Gepotidacin MIC_90_s against the Enterobacterales species tested were 4 µg/mL for *E. coli* (1,000) and *Klebsiella oxytoca* (250), 8 µg/mL for *Citrobacter* spp. (250) and *Klebsiella aerogenes* (250), 16 µg/mL for *Proteus mirabilis* (250) and *Providencia rettgeri* (250), and 32 µg/mL for *Enterobacter cloacae* (500) and *K. pneumoniae* (500). MIC ranges and percent of isolates with MICs ≤ 16 µg/mL for each species were as follows: *E. coli* (0.12–64 µg/mL; 99.8), *Citrobacter* spp. (0.5–128 µg/mL; 98.8), *K. pneumoniae* (0.5 to >128 µg/mL; 88.6), *E. cloacae* (1 to >128 µg/mL; 86.6), *K. aerogenes* (0.06–64 µg/mL; 97.6), *K. oxytoca* (0.5–64 µg/mL; 99.2), *P. mirabilis* (0.25–128 µg/mL; 91.2), *P. rettgeri* (0.5 to >128 µg/mL; 93.2) (Tables 1 and 2). Twenty-one Enterobacterales isolates exhibited gepotidacin MIC values of 128 µg/mL (six *E. cloacae*, six *K. pneumoniae*, five *P. rettgeri*, three *P. mirabilis*, and one *Citrobacter* spp.). isolate), and eight isolates had MIC values of >128 µg/mL (three *E. cloacae*, three *P. rettgeri*, and two *K. pneumoniae*) (Table 2; Table S1).

**TABLE 1:** *In vitro* activity of gepotidacin and comparator agents against Enterobacterales

		MIC (µg/mL)			MIC interpretation^*a*^		
Organism (no. of isolates)	Antimicrobial agent	MIC_50_	MIC_90_	MIC range	% susceptible	% intermediate	% resistant
*Citrobacter* spp.^*b*^ (250)	Gepotidacin	2	8	0.5–128	NA^*c*^	NA	NA
	Ceftazidime	0.25	>16	0.06 to >16	82.4	0.8	16.8
	Ciprofloxacin	0.015	0.25	0.002 to >128	90.4	1.6	8.0
	Doxycycline	2	8	0.5 to >16	88.4	2.4	9.2
	Fosfomycin	2	8	0.25–32	NA	NA	NA
	Meropenem	0.03	0.06	0.015 to >8	99.2	0	0.8
	Nitrofurantoin	32	64	≤0.5 to >128	72.0	23.2	4.8
	Piperacillin-tazobactam	4	32	0.5 to >128	86.0	9.6	4.4
	Trimethoprim-sulfamethoxazole	0.12	4	≤0.06 to >4	89.6	NA	10.4
*Enterobacter cloacae* (500)	Gepotidacin	8	32	1 to >128	NA	NA	NA
	Ceftazidime	1	>16	0.06 to >16	59.2	1.2	39.6
	Ciprofloxacin	0.015	8	0.002 to >128	74.8	4.4	20.8
	Doxycycline	4	>16	1 to >16	75.6	6.4	18.0
	Fosfomycin	64	256	0.5 to >256	NA	NA	NA
	Meropenem	0.06	0.25	0.015 to >8	95.0	0.8	4.2
	Nitrofurantoin	64	128	1 to >128	17.2	35.8	47.0
	Piperacillin-tazobactam	4	>128	1 to >128	66.2	8.8	25.0
	Trimethoprim-sulfamethoxazole	0.25	>4	≤0.06 to >4	70.6	NA	29.4
*Escherichia coli* (1,000)	Gepotidacin	2	4	0.12–64	NA	NA	NA
	Ceftazidime	0.25	>16	≤0.03 to >16	81.8	3.3	14.9
	Ciprofloxacin	0.015	64	0.004 to >128	64.8	2.8	32.4
	Doxycycline	2	>16	0.25 to >16	65.1	12.4	22.5
	Fosfomycin	2	16	0.5 to >256	98.0	0.8	1.2
	Meropenem	0.03	0.03	≤0.004 to >8	99.5	0	0.5
	Nitrofurantoin	16	16	≤0.5 to >128	96.3	2.0	1.7
	Piperacillin-tazobactam	2	8	≤0.12 to >128	95.7	2.3	2.0
	Trimethoprim-sulfamethoxazole	0.12	>4	≤0.06 to >4	63.4	NA	36.6
*Klebsiella aerogenes* (250)	Gepotidacin	4	8	0.06–64	NA	NA	NA
	Ceftazidime	0.25	>16	0.06 to >16	80.4	0.4	19.2
	Ciprofloxacin	0.015	0.12	0.004–128	91.2	0.4	8.4
	Doxycycline	2	4	0.5 to >16	90.8	1.2	8.0
	Fosfomycin	64	128	1 to >256	NA	NA	NA
	Meropenem	0.06	0.06	0.015 to >8	98.4	0	1.6
	Nitrofurantoin	64	128	8 to >128	19.2	40.4	40.4
	Piperacillin-tazobactam	4	64	0.25 to >128	84.4	10.0	5.6
	Trimethoprim-sulfamethoxazole	0.12	0.5	≤0.06 to >4	95.2	NA	4.8
*Klebsiella oxytoca* (250)	Gepotidacin	4	4	0.5–64	NA	NA	NA
	Ceftazidime	0.12	4	≤0.03 to >16	90.4	2.8	6.8
	Ciprofloxacin	0.015	1	0.004–16	87.2	2.0	10.8
	Doxycycline	1	8	0.5 to >16	86.8	4.8	8.4
	Fosfomycin	32	256	2 to >256	NA	NA	NA
	Meropenem	0.03	0.06	0.015 to >8	99.2	0	0.8
	Nitrofurantoin	32	64	≤0.5 to >128	84.8	12.4	2.8
	Piperacillin-tazobactam	2	>128	0.5 to >128	86.4	0.8	12.8
	Trimethoprim-sulfamethoxazole	0.12	>4	≤0.06 to >4	87.2	NA	12.8
*Klebsiella pneumoniae* (500)	Gepotidacin	8	32	0.5 to >128	NA	NA	NA
	Ceftazidime	0.25	>16	≤0.03 to >16	73.6	1.0	25.4
	Ciprofloxacin	0.03	64	0.008 to >128	67.6	6.4	26.0
	Doxycycline	2	>16	1 to >16	68.2	4.6	27.2
	Fosfomycin	64	>256	1 to >256	NA	NA	NA
	Meropenem	0.03	0.12	0.015 to >8	92.4	1.0	6.6
	Nitrofurantoin	128	>128	2 to >128	23.2	21.6	55.2
	Piperacillin-tazobactam	4	>128	0.5 to >128	81.0	6.0	13.0
	Trimethoprim-sulfamethoxazole	0.25	>4	≤0.06 to >4	69.4	NA	30.6
*Proteus mirabilis* (250)	Gepotidacin	8	16	0.25–128	NA	NA	NA
	Ceftazidime	0.06	1	≤0.03 to >16	93.6	1.2	5.2
	Ciprofloxacin	0.03	32	0.015 to >128	66.4	1.6	32.0
	Doxycycline	>16	>16	4 to >16	1.2	0	98.8
	Meropenem	0.06	0.12	0.03 to >8	98.4	0	1.6
	Nitrofurantoin	128	128	32 to >128	0.8	6.4	92.8
	Piperacillin-tazobactam	0.5	1	≤0.12 to 128	98.8	0.8	0.4
	Trimethoprim-sulfamethoxazole	0.25	>4	≤0.06 to >4	62.8	NA	37.2
*Providencia rettgeri* (250)	Gepotidacin	8	16	0.5 to >128	NA	NA	NA
	Ceftazidime	0.06	0.5	≤0.03 to >16	96.0	0.4	3.6
	Ciprofloxacin	0.03	4	0.008 to >128	80.8	4.0	15.2
	Doxycycline	>16	>16	1 to >16	1.2	12.8	86.0
	Fosfomycin	128	>256	2 to >256	NA	NA	NA
	Meropenem	0.06	0.12	0.015 to >8	97.2	0	2.8
	Nitrofurantoin	128	>128	4 to >128	6.0	4.0	90.0
	Piperacillin-tazobactam	0.5	2	≤0.12 to >128	97.2	1.2	1.6
	Trimethoprim-sulfamethoxazole	0.25	>4	0.12 to >4	80.8	NA	19.2

^*a*^ Based on CLSI M100 2021 breakpoints.

^*b*^ *Citrobacter* spp. isolates comprised the following: *Citrobacter amalonaticus* (*n* = 7), *Citrobacter braakii* (*n* = 7), *Citrobacter farmeri* (*n* = 2), *Citrobacter freundii* (*n* = 126), *Citrobacter koseri* (*n* = 106), *Citrobacter murliniae* (*n* = 1), and *Citrobacter youngae* (*n* = 1).

^*c*^ NA, not available. CLSI M100 MIC breakpoints are not published for this antimicrobial agent.

**TABLE 2:** Distribution of gepotidacin MICs against Enterobacterales and *E. faecalis* including isolates not susceptible^*a*^ to ciprofloxacin or presumptive ESBL-producing *E. coli* and *K. pneumoniae*^*b*,*c*^

	Gepotidacin MIC (µg/mL)													
Organism	Cumulative percent of isolates inhibited by MIC (no. of isolates with MIC)^*e*^													
Organism or phenotype (no.)	≤0.03	0.06	0.12	0.25	0.5	1	2	4	8	16	32	64	128	>128
Enterobacterales (3,250)		0.1 (1)	0.1 (3)	0.3 (5)	1.5 (41)	8.6 (230)	38.4 (967)	69.0 (995)	86.1 (557)	**94.6^*d*^** **(275)**	97.7 (100)	99.1 (47)	99.8 (21)	100 (8)
Ciprofloxacin not susceptible (850)				0.6 (5)	3.3 (23)	13.9 (90)	36.6 (193)	56.4 (168)	69.6 (113)	82.8 (112)	**92.1** (**79**)	97.1 (42)	99.3 (19)	100 (6)
*Citrobacter* spp. (250)					1.2 (3)	23.2 (55)	70 (117)	88.8 (47)	**95.6** (**17**)	98.8 (8)	99.6 (2)	99.6 (0)	100 (1)	
Ciprofloxacin not susceptible (24)						8.3 (2)	25.0 (4)	58.3 (8)	83.3 (6)	**91.7** (**2**)	95.8 (1)	95.8 (0)	100 (1)	
*Enterobacter cloacae* (500)						0.4 (2)	5.8 (27)	47 (206)	73.4 (132)	86.6 (66)	**93.2** (**33**)	98.2 (25)	99.4 (6)	100 (3)
Ciprofloxacin not susceptible (126)						0.8 (1)	4.0 (4)	14.3 (13)	33.3 (24)	56.3 (29)	77.0 (26)	**93.7** (**21**)	98.4 (6)	100 (2)
*Escherichia coli* (1,000)			0.2 (2)	0.6 (4)	3.6 (30)	17.5 (139)	75.0 (575)	**95.3** (**203**)	97.7 (24)	99.8 (21)	99.9 (1)	100 (1)		
Ciprofloxacin not susceptible (352)				1.1 (4)	6.2 (18)	24.1 (63)	68.5 (156)	**92.9** (**86**)	96.0 (11)	99.4 (12)	99.7 (1)	100 (1)		
Ceftazidime ≥ 2 µg/mL (228)				1.3 (3)	5.7 (10)	20.2 (33)	68.4 (110)	89.9 (49)	**95.2** (**12**)	100 (11)				
*Klebsiella aerogenes* (250)		0.4 (1)	0.8 (1)	(0)	1.2 (1)	4.4 (8)	42.4 (95)	88.8 (116)	**94.8** (**15**)	97.6 (7)	99.6 (5)	100 (1)		
Ciprofloxacin not susceptible (22)						9.1 (2)	13.6 (1)	27.3 (3)	59.1 (7)	72.7 (3)	**95.5** (**5**)	100 (1)		
*Klebsiella oxytoca* (250)					0.4 (1)	5.2 (12)	48.4 (108)	**91.6** (**108**)	97.2 (14)	99.2 (5)	99.6 (1)	100 (1)		
Ciprofloxacin not susceptible (32)						31.2 (10)	50 (6)	62.5 (4)	84.4 (7)	**93.8** (**3**)	96.9 (1)	100 (1)		
*Klebsiella pneumoniae* (500)					0.4 (2)	1.4 (5)	4.6 (16)	40.2 (178)	75.2 (175)	88.6 (67)	**96.6** (**40**)	98.4 (9)	99.6 (6)	100 (2)
Ciprofloxacin not susceptible (162)					0.6 (1)	2.5 (3)	8.0 (9)	19.8 (19)	42.0 (36)	68.5 (43)	89.5 (34)	**95.1** (**9**)	98.8 (6)	100 (2)
Ceftazidime ≥ 2 µg/mL (145)						2.1 (3)	7.6 (8)	22.1 (21)	49.7 (40)	77.2 (40)	**91.7** (**21**)	95.9 (6)	99.3 (5)	100 (1)
*Proteus mirabilis* (250)				0.4 (1)	1.2 (2)	2.8 (4)	7.6 (12)	22.8 (38)	63.6 (102)	**91.2** (**69**)	96.4 (13)	98.8 (6)	100 (3)	
Ciprofloxacin not susceptible (84)				1.2 (1)	3.6 (2)	8.3 (4)	22.6 (12)	54.8 (27)	69.0 (12)	81.0 (10)	**90.5** (**8**)	96.4 (5)	100 (3)	
*Providencia rettgeri* (250)					0.8 (2)	2.8 (5)	9.6 (17)	49.2 (99)	80.4 (78)	**93.2** (**32**)	95.2 (5)	96.8 (4)	98.8 (5)	100 (3)
Ciprofloxacin not susceptible (48)					4.2 (2)	14.6 (5)	16.7 (1)	33.3 (8)	54.2 (10)	75 (10)	81.2 (3)	89.6 (4)	**95.8** (**3**)	100 (2)
*Enterococcus faecalis* (500)				0.4 (2)	5.2 (24)	22.2 (85)	84.4 (311)	**99.2** (**74**)	99.6 (2)	99.8 (1)	99.8 (0)	100 (1)		
Ciprofloxacin not susceptible (149)				0.7 (1)	12.8 (18)	55.0 (63)	85.9 (46)	**98.0** (**18**)	99.3 (2)	100 (1)				

^*a*^ Susceptibility based on CLSI M100 2021.

^*b*^ Presumptive ESBL-producing isolates of *E. coli* and *K. pneumoniae* defined by a ceftazidime MIC ≥ 2 µg/mL.

^*c*^ There were no ciprofloxacin-non-susceptible isolates of *Staphylococcus saprophyticus* identified.

^*d*^ MIC_90_ is in boldface for each MIC distribution.

^*e*^ Empty cells show there are no isolates which have the MIC.

In total, 26.2% (850/3,250) of all isolates of Enterobacterales tested were ciprofloxacin-not susceptible (Table 2). Gepotidacin MIC_50/90_ was 4/32 µg/mL against all ciprofloxacin not susceptible Enterobacterales isolates. Gepotidacin MIC_90_s for ciprofloxacin not susceptible *E. coli* (352/1,000), *Citrobacter* spp. (24/250), *K. pneumoniae* (162/500), *E. cloacae* (126/500), *K. aerogenes* (22/250), *K. oxytoca* (32/250), *P. mirabilis* (84/250), and *P. rettgeri* (48/250) isolates were 4, 16, 64, 64, 32, 16, 32, and 128 µg/mL, respectively (Tables 2 and 3).

**TABLE 3:** *In vitro* activity of gepotidacin and comparator agents against ciprofloxacin not susceptible Enterobacterales

		MIC (µg/mL)			MIC interpretation^*a*^		
Organism (no. of isolates)	Antimicrobial agent	MIC_50_	MIC_90_	MIC range	% susceptible	% intermediate	% resistant
*Citrobacter* spp.^*b*^ (24)	Gepotidacin	4	16	1–128	NA^*c*^	NA	NA
	Ceftazidime	2	>16	0.06 to >16	62.5	4.2	33.3
	Ciprofloxacin	4	64	0.5 to >128	0	16.7	83.3
	Doxycycline	16	>16	2 to >16	33.3	4.2	62.5
	Fosfomycin	2	16	0.5–32	NA	NA	NA
	Meropenem	0.03	1	0.015 to >8	91.7	0	8.3
	Nitrofurantoin	16	128	8 to >128	70.8	8.3	20.8
	Piperacillin-tazobactam	8	>128	0.5 to >128	70.8	8.3	20.8
	Trimethoprim-sulfamethoxazole	0.5	>4	≤0.06 to >4	50	NA	50
*Enterobacter cloacae* (126)	Gepotidacin	16	64	1 to >128	NA	NA	NA
	Ceftazidime	>16	>16	0.25 to >16	26.2	2.4	71.4
	Ciprofloxacin	4	128	0.5 to >128	0	17.5	82.5
	Doxycycline	8	>16	2 to >16	43.7	14.3	42.1
	Fosfomycin	64	256	2 to >256	NA	NA	NA
	Meropenem	0.06	>8	0.015 to >8	81.8	2.4	15.9
	Nitrofurantoin	128	>128	4 to >128	9.5	27.8	62.7
	Piperacillin-tazobactam	32	>128	2 to >128	46	11.9	42.1
	Trimethoprim-sulfamethoxazole	>4	>4	≤0.06 to >4	20.6	NA	79.4
*Escherichia coli* (352)	Gepotidacin	2	4	0.25–64	NA	NA	NA
	Ceftazidime	1	>16	≤0.03 to >16	58.8	6.8	34.4
	Ciprofloxacin	32	128	0.5 to >128	0	8	92.1
	Doxycycline	8	>16	0.5 to >16	44	19.3	36.7
	Fosfomycin	2	16	0.5 to >256	97.4	0.9	1.7
	Meropenem	0.03	0.06	0.015 to >8	98.9	0	1.1
	Nitrofurantoin	16	32	≤0.5 to >128	93.8	2.6	3.7
	Piperacillin-tazobactam	4	16	≤0.12 to >128	90.3	4.6	5.1
	Trimethoprim-sulfamethoxazole	>4	>4	≤0.06 to >4	42.1	NA	58
*Klebsiella aerogenes* (22)	Gepotidacin	8	32	1–64	NA	NA	NA
	Ceftazidime	16	>16	0.25 to >16	36.4	4.6	59.1
	Ciprofloxacin	1	32	0.5–128	0	4.6	95.5
	Doxycycline	4	16	1 to >16	63.6	0	36.4
	Fosfomycin	32	256	2 to >256	NA	NA	NA
	Meropenem	0.03	0.5	0.015 to >8	90.9	0	9.1
	Nitrofurantoin	64	128	8 to >128	31.8	22.7	45.5
	Piperacillin-tazobactam	16	64	2 to >128	59.1	31.8	9.1
	Trimethoprim-sulfamethoxazole	0.5	>4	≤0.06 to >4	54.6	NA	45.5
*Klebsiella oxytoca* (32)	Gepotidacin	2	16	1–64	NA	NA	NA
	Ceftazidime	8	>16	0.06 to >16	43.8	15.6	40.6
	Ciprofloxacin	2	8	0.5–16	0	15.6	84.4
	Doxycycline	8	16	0.5 to >16	40.6	18.8	40.6
	Fosfomycin	32	256	8 to >256	NA	NA	NA
	Meropenem	0.03	0.06	0.015 to >8	96.9	0	3.1
	Nitrofurantoin	16	64	4–128	84.4	12.5	3.1
	Piperacillin-tazobactam	8	>128	1 to >128	62.5	3.1	34.4
	Trimethoprim-sulfamethoxazole	>4	>4	≤0.06 to >4	40.6	NA	59.4
*Klebsiella pneumoniae* (162)	Gepotidacin	16	64	0.5 to >128	NA	NA	NA
	Ceftazidime	>16	>16	0.06 to >16	25.9	3.1	71
	Ciprofloxacin	16	>128	0.5 to >128	0	19.8	80.3
	Doxycycline	16	>16	1 to >16	28.4	10.5	61.1
	Fosfomycin	64	>256	8 to >256	NA	NA	NA
	Meropenem	0.06	>8	0.015 to >8	79	3.1	17.9
	Nitrofurantoin	128	>128	16 to >128	10.5	16.1	73.5
	Piperacillin-tazobactam	16	>128	1 to >128	52.5	15.4	32.1
	Trimethoprim-sulfamethoxazole	>4	>4	≤0.06 to >4	22.8	NA	77.2
*Proteus mirabilis* (84)	Gepotidacin	4	32	0.25–128	NA	NA	NA
	Ceftazidime	0.12	>16	≤0.03 to >16	84.5	1.2	14.3
	Ciprofloxacin	16	>128	0.5 to >128	0	4.8	95.2
	Doxycycline	>16	>16	4 to >16	1.2	0	98.8
	Meropenem	0.06	0.12	0.03 to >8	96.4	0	3.6
	Nitrofurantoin	128	>128	32 to >128	1.2	8.3	90.5
	Piperacillin-tazobactam	0.5	2	≤0.12 to 64	97.6	2.4	0
	Trimethoprim-sulfamethoxazole	>4	>4	0.12 to >4	21.4	NA	78.6
*Providencia rettgeri* (48)	Gepotidacin	8	128	0.5 to >128	NA	NA	NA
	Ceftazidime	0.12	>16	≤0.03 to >16	87.5	0	12.5
	Ciprofloxacin	4	>128	0.5 to >128	0	20.8	79.2
	Doxycycline	>16	>16	2 to >16	4.2	6.3	89.6
	Fosfomycin	256	>256	4 to >256	NA	NA	NA
	Meropenem	0.06	8	0.03 to >8	87.5	0	12.5
	Nitrofurantoin	>128	>128	16 to >128	2.1	10.4	87.5
	Piperacillin-tazobactam	1	32	0.25 to >128	87.5	4.2	8.3
	Trimethoprim-sulfamethoxazole	4	>4	0.12 to >4	39.6	0	60.4

^*a*^ Based on CLSI M100 2021 breakpoints.

^*b*^ *Citrobacter* spp. isolates comprised *Citrobacter freundii* (*n* = 22) and *Citrobacter koseri* (*n* = 2).

^*c*^ NA, not available. CLSI M100 MIC breakpoints are not published for this antimicrobial agent.

The MIC_90_s for gepotidacin against 228 (22.8%) isolates of *E. coli* and 145 (29%) isolates of *K. pneumoniae* with ceftazidime MICs of ≥2 µg/mL (presumptive ESBLs) were 8 and 32 µg/mL, respectively (Table 4). Among presumptive ESBL-producing *E. coli* and *K. pneumoniae*, 25.0% and 13.8%, respectively, were ciprofloxacin susceptible (*E. coli* ciprofloxacin MIC_90_, 128 µg/mL; *K. pneumoniae* ciprofloxacin MIC_90_, >128 µg/mL).

**TABLE 4:** *In vitro* activity of gepotidacin and comparator agents against presumptive ESBL-producing *E. coli* and *K. pneumoniae*^*a*^

		MIC (µg/mL)			MIC interpretation^*b*^		
Organism (no. of isolates)	Antimicrobial agent	MIC_50_	MIC_90_	MIC range	% susceptible	% intermediate	% resistant
*Escherichia coli* (228)	Gepotidacin	2	8	0.25–16	NA^*c*^	NA	NA
	Ceftazidime	16	>16	2 to >16	20.2	14.5	65.4
	Ciprofloxacin	32	128	0.008 to >128	25.0	2.2	72.8
	Doxycycline	8	>16	0.25 to >16	41.2	22.8	36.0
	Fosfomycin	2	16	0.5 to >256	96.5	1.3	2.2
	Meropenem	0.03	0.06	0.015 to >8	97.8	0	2.2
	Nitrofurantoin	8	32	≤0.5 to 128	93.0	4.0	3.1
	Piperacillin-tazobactam	4	32	≤0.12 to >128	87.7	6.6	5.7
	Trimethoprim-sulfamethoxazole	>4	>4	≤0.06 to >4	40.4	NA	59.7
*Klebsiella pneumoniae* (145)	Gepotidacin	16	32	1 to >128	NA	NA	NA
	Ceftazidime	>16	>16	2 to >16	9.0	3.5	87.6
	Ciprofloxacin	16	>128	0.008 to >128	13.8	8.3	77.9
	Doxycycline	16	>16	1 to >16	29.7	9.0	61.4
	Fosfomycin	64	>256	8 to >256	NA	NA	NA
	Meropenem	0.06	>8	0.015 to >8	73.8	3.5	22.8
	Nitrofurantoin	128	>128	2 to >128	10.4	17.9	71.7
	Piperacillin-tazobactam	32	>128	1 to >128	41.4	17.9	40.7
	Trimethoprim-sulfamethoxazole	>4	>4	≤0.06 to >4	22.1	NA	77.9

^*a*^ Presumptive ESBL-producing isolates of *E. coli* and *K. pneumoniae* defined by a ceftazidime MIC ≥ 2 µg/mL.

^*b*^ Based on CLSI M100 2021 breakpoints.

^*c*^ NA, not available. CLSI M100 MIC breakpoints are not published for this antimicrobial agent.

The MIC_50_ and MIC_90_ for gepotidacin against 500 *E. faecalis* isolates were 2 and 4 µg/mL, respectively (Tables 2 and 5; Table S1). The MIC_50_ and MIC_90_ for gepotidacin against the subset of ciprofloxacin not susceptible *E. faecalis* (149/500) were 1 and 4 µg/mL, respectively.

**TABLE 5:** *In vitro* activity of gepotidacin and comparator agents against *E. faecalis* and *S. saprophyticus*

		MIC (µg/mL)			MIC interpretation^*a*^		
Organism (no. of isolates)	Antimicrobial agent	MIC_50_	MIC_90_	MIC range	% susceptible	% intermediate	% resistant
*Enterococcus faecalis* (500)	Gepotidacin	2	4	0.25–64	NA^*b*^	NA	NA
	Ceftazidime	>16	>16	4 to >16	NA	NA	NA
	Ciprofloxacin	1	64	0.03 to >128	70.2	3.2	26.6
	Doxycycline	8	8	≤0.06 to >16	33.0	59.0	8.0
	Fosfomycin	64	128	≤0.06 to 256	88.4	10.2	1.4
	Meropenem	4	8	0.06 to >8	NA	NA	NA
	Nitrofurantoin	8	16	≤0.5 to 128	99.0	0.4	0.6
	Piperacillin-tazobactam	4	8	1 to >128	NA	NA	NA
	Trimethoprim-sulfamethoxazole	≤0.06	>4	≤0.06 to >4	NA	NA	NA
*Enterococcus faecalis* (149)	Gepotidacin	1	4	0.25–16	NA	NA	NA
Ciprofloxacin not susceptible	Ceftazidime	>16	>16	16 to >16	NA	NA	NA
	Ciprofloxacin	64	128	2 to >128	0	10.7	89.3
	Doxycycline	8	8	0.12 to >16	26.9	65.8	7.4
	Fosfomycin	64	128	1–256	85.2	12.1	2.7
	Meropenem	4	8	1 to >8	NA	NA	NA
	Nitrofurantoin	8	16	4–64	99.3	0.7	0
	Piperacillin-tazobactam	8	32	2 to >128	NA	NA	NA
	Trimethoprim-sulfamethoxazole	>4	>4	≤0.06 to >4	NA	NA	NA
*Staphylococcus saprophyticus* (250)	Gepotidacin	0.12	0.12	0.06–2	NA	NA	NA
	Ceftazidime	>16	>16	8 to >16	NA	NA	NA
	Ciprofloxacin	0.25	0.5	0.25–1	100	0	0
	Doxycycline	0.25	1	0.12–4	100	0	0
	Fosfomycin	128	>256	32 to >256	NA	NA	NA
	Meropenem	0.25	0.25	0.06–8	NA	NA	NA
	Nitrofurantoin	16	16	4–128	98.4	0.8	0.8
	Piperacillin-tazobactam	2	4	0.25 to >128	NA	NA	NA
	Trimethoprim-sulfamethoxazole	≤0.06	0.12	≤0.06 to >4	96.4	NA	3.6

^*a*^ Based on CLSI M100 2021 breakpoints.

^*b*^ NA, not available. CLSI M100 MIC breakpoints are not published for this antimicrobial agent.

The MIC_50_ and MIC_90_ for gepotidacin against 250 *S*. *saprophyticus* isolates were both 0.12 µg/mL. All isolates of *S. saprophyticus* were susceptible to ciprofloxacin (Table 5; Table S1).

The MIC_50_ and MIC_90_ for gepotidacin against the 50 isolates chosen for MBC testing were 4 and 8 µg/mL, respectively (Table 6). The MBC_50_ and MBC_90_ for gepotidacin against the 50 isolates were 4 and 16 µg/mL, respectively (Table 6). Gepotidacin was bactericidal against the majority of isolates tested, with a total of 94% (47/50) of isolates exhibiting an MBC/MIC ratio of ≤4 (Enterobacterales [92.5%, 37/40], *E. faecalis* [100%, 5/5], and *S. saprophyticus* [100%, 5/5]) (Table S2). For 92% (46/50) of the isolates tested, the gepotidacin MBC/MIC ratio was ≤2 (Enterobacterales [90%, 36/40], *E. faecalis* [100%, 5/5], and *S. saprophyticus* [100%, 5/5]). Gepotidacin was not bactericidal against three Enterobacterales isolates; one isolate each of *E. coli* (MBC/MIC of 8), *E. cloacae* (MBC/MIC of ≥64), and *K. oxytoca* (MBC/MIC of ≥64).

**TABLE 6:** Summary of MIC_50/90_ values, MBC_50/90_ values, and MBC/MIC ratios for gepotidacin against 50 isolates

Measure	MIC or MBC (µg/mL)	
	50%	90%
MIC	4	8
MBC	4	16
MBC/MIC ratio	1	2

## DISCUSSION

Previous studies describing the *in vitro* activity of gepotidacin show that it is active against fastidious and non-fastidious, aerobic and anaerobic gram-positive and gram-negative bacteria, including isolates resistant to fluoroquinolones and agents of other antimicrobial classes (13–18). In the current study, gepotidacin was shown to be active *in vitro* against 4,000 clinical isolates (predominantly UTI isolates) of Enterobacterales, *E. faecalis*, and *S. saprophyticus*. Gepotidacin activity against ciprofloxacin not susceptible and presumptive ESBL-positive subpopulations was broadly similar to its activity against respective total populations, demonstrating the robustness of gepotidacin activity against commonly occurring resistant strains. Gepotidacin MIC_90_/MIC_90CIP-NS_ values were 4/4, 4/16, 8/16, 8/32, 16/32, 16/128, 32/64, and 32/64 µg/mL for *E. coli*, *K. oxytoca*, *Citrobacter* spp., *K. aerogenes*, *P. mirabilis*, *P. rettgeri*, *E. cloacae*, and *K. pneumoniae*, respectively (Table 2). Gepotidacin MIC_90_s for *E. coli* and *K. pneumoniae* presumptive ESBL producers were 8 and 32 µg/mL, respectively (Tables 2 and 4). Regarding Gram positives, gepotidacin MIC_90_/MIC_90CIP-NS_ values for *E. faecalis* were 4/4 µg/mL, and the MIC_90_ for *S. saprophyticus* was 0.12 µg/mL (no *S. saprophyticus* ciprofloxacin resistance was observed in this study) (Tables 2 and 5). As expected, gepotidacin was bactericidal against most isolates tested, with an MBC/MIC ratio ≤4 against 94% of all isolates tested (Enterobacterales [92.5%], *E. faecalis* [100%], and *S. saprophyticus* [100%]) (Table 6; Table S2).

This is the first manuscript reporting gepotidacin MIC_50_s, MIC_90_s, and MBC/MIC ratios for uropathogens other than *E. coli* and *S. saprophyticus*. In terms of validation, Flamm et al. previously reported a gepotidacin MIC_90_ of 4 µg/mL for *E. coli* (*n* = 25) (14), agreeing with the MIC_90_ generated in the current study for *E. coli*. Flamm et al. also reported that gepotidacin was bactericidal against *E. coli*, with MBC/MIC ratios of ≤4 against 88% of the isolates tested, respectively, again similar to our finding for *E. coli* (and other Enterobacterales); and that gepotidacin bactericidal activity (assessed by time-kill curves) was observed at 24 h with exposure to 4× or 10× MIC (14). Biedenbach et al. tested 1,010 *E. coli* isolates and reported a gepotidacin MIC_90_ of 4 µg/mL for both levofloxacin not susceptible *E. coli* (*n* = 278) and ESBL-positive *E. coli* (*n* = 179) isolate subsets (13), similar to our observations and those of Flamm et al. (14). And most recently, data from a global prospective surveillance study (2019–2020) were also similar to our observations with reported gepotidacin MIC_90_ values of 2 µg/mL for *E. coli* (*n* = 3,560) and 0.12 µg/mL for *S. saprophyticus* (*n* = 344) (18).

There are limitations to the results generated by the current study. First, the study did not test amoxicillin-clavulanate and cefazolin (cephalexin surrogate), oral treatments for uUTIs, and therefore, those comparative data are not available for review in the context of the MIC results generated for the other first-line and second-line agents tested. Second, confirmatory phenotypic or molecular ESBL testing was not performed; therefore, some isolates of *E. coli* and *K. pneumoniae* presumptively identified as ESBL-producers may have been truly susceptible or may have carried carbapenemases or expressed other resistance mechanisms (outer membrane protein [OMP] changes, efflux). Third, gepotidacin resistance mechanisms were not investigated in rare isolates with higher gepotidacin MICs (≥128 µg/mL). The characterization of these isolates will be the subject of a future publication.

In the current study, gepotidacin demonstrated *in vitro* activity against a robust collection of Enterobacterales, *E. faecalis*, and *S. saprophyticus*, including many isolates resistant to ciprofloxacin and agents of other classes. Because of its distinct binding site and unique mode of action, gepotidacin is active *in vitro* against most target pathogens of UTI presumptively carrying determinants of resistance to established antibacterial agents, including fluoroquinolones. Our *in vitro* data support continued development of gepotidacin for the treatment of uUTIs.

## MATERIALS AND METHODS

### Bacterial isolates

The 4,000 bacterial isolates tested in this study were randomly selected from the culture collection maintained by IHMA (Schaumburg, USA). The isolates were collected by IHMA as part of global surveillance studies across seven regions (Table S3) from 2012 to 2020 (Table S4). The majority of isolates were collected in North America (59.2%) and Europe (22.3%) in 2019 (70.4%) and 2020 (14.7%) (Tables S3 and S4). The identities of all isolates were confirmed at IHMA using matrix-assisted laser desorption ionization-time of flight mass spectrometry (Bruker Daltonics, Billerica, USA). Isolates were limited to one per patient and were preferentially selected from UTI sources (3,980 isolates; 99.5% of all isolates tested); other sources included skin and soft tissue infection (nine isolates, all *P. rettgeri*; 0.2% of all isolates tested), bloodstream infection (five isolates comprising four *P. rettgeri* and one *E. faecalis*; 0.1%), intra-abdominal infection (four isolates, all *P. rettgeri*; 0.1%), and respiratory tract infection (two isolates, both *P. rettgeri*; <0.1%). Study isolates comprised 3,250 gram-negative bacilli (all Enterobacterales) and 750 gram-positive cocci (500 *E. faecalis* and 250 *S*. *saprophyticus*). The composition of Enterobacterales isolates by species is shown in Tables S3 and S4.

MBC study isolates included five randomly chosen isolates from each of the 10 bacterial species included in the 4,000-isolate collection (i.e., five isolates of each of *C. koseri*, *E. cloacae*, *E. faecalis*, *E. coli*, *K. aerogenes*, *K. oxytoca*, *K. pneumoniae*, *P. mirabilis*, *P. rettgeri*, and *S. saprophyticus*) (Table S2).

### Antimicrobial susceptibility testing

Gepotidacin (lot number 192416007) was provided by GSK (Collegeville, PA, USA). Piperacillin and sulfamethoxazole were purchased from Sigma-Aldrich, Inc. (St. Louis, MO, USA). All other antimicrobial powders were obtained from United States Pharmacopeia (Rockville, MD, USA). All antimicrobial agents were dissolved and diluted following CLSI instructions (20). MICs were determined in cation-adjusted Mueller-Hinton broth by broth microdilution following the CLSI standard method (21) for all compounds except fosfomycin. Fosfomycin MICs were determined by agar dilution following the CLSI standard method (21). Inoculation of MIC panels and agar dilution plates from common inocula took place within 15 minutes of adjustment of inocula suspension turbidities. The panels and agar dilution plates were incubated at 35 ± 2°C in an ambient atmosphere for 16–20 hours before reading the MIC endpoints (20). Quality control testing was performed each day of testing as specified by CLSI using *E. coli* ATCC 25922, *Pseudomonas aeruginosa* ATCC 27853, *E. faecalis* ATCC 29212, and *Staphylococcus aureus* ATCC 29213 (20). Data were analyzed using CLSI 2021 interpretative criteria (6). MIC interpretative (breakpoint) criteria are not yet available for gepotidacin.

### Minimum bactericidal concentration testing

MBC testing was performed for gepotidacin for 50 study isolates following the M26-A CLSI guideline (22). A baseline colony count was determined from the inoculum of the broth microdilution panel. Following incubation of the broth microdilution panel, 10 µL of each well showing no visible growth within the doubling-dilution series for gepotidacin was plated to a blood agar plate (exception: MacConkey agar was used for *P. mirabilis* to prevent swarming and permit accurate colony counts). The last well of growth in the drug dilution series was also sub-cultured as a purity control for the isolate being tested. Following 24 hours of incubation, the colonies observed for each dilution were counted. The MBC was defined as a ≥3 log_10_ (≥99.9%) decrease in the CFU/mL count compared to the starting inoculum (22).

## SUPPLEMENTAL MATERIAL

Supplemental material aac.00296-25-s0001.docx Tables S1 to S4.

ASM does not own the copyrights to Supplemental Material that may be linked to, or accessed through, an article. The authors have granted ASM a non-exclusive, world-wide license to publish the Supplemental Material files. Please contact the corresponding author directly for reuse.
