# Supplementary material for: In vitro activity of gepotidacin against urinary tract infection isolates of Enterobacterales, Enterococcus faecalis, and Staphylococcus saprophyticus
Source: Antimicrob Agents Chemother. 2025 May 15;69(6):e00296-25. doi: 10.1128/aac.00296-25 (PMC12135502; doi:10.1128/aac.00296-25)
Supplement: Supplemental material — Tables S1 to S4. [file aac.00296-25-s0001.docx]

**TABLE S1** Distribution of gepotidacin and ciprofloxacin MICs against Enterobacterales, *E. faecalis*, and *S. saprophyticus*

| Organism (no. of isolates) | MIC (µg/mL)  Cumulative percent of isolates inhibited by MIC  (no. of isolates with MIC)^a^ | | | | | | | | | | | | |
| --- | --- | --- | --- | --- | --- | --- | --- | --- | --- | --- | --- | --- | --- |
| Antimicrobial agent | ≤0.03 | 0.06 | 0.12 | 0.25 | 0.5 | 1 | 2 | 4 | 8 | 16 | 32 | 64 | ≥128 |
| *Citrobacter* spp. (250) |  |  |  |  |  |  |  |  |  |  |  |  |  |
| Gepotidacin |  |  |  |  | 1.2 | 23.2 | 70.0 | 88.8 | **95.6** | 98.8 | 99.6 | 99.6 | 100 |
|  |  |  |  |  | (3) | (55) | (117) | (47) | **(17)** | (8) | (2) | (0) | (1) |
| Ciprofloxacin | 74.0 | 82.0 | 88.0 | **90.4** | 92.0 | 93.2 | 94.0 | 95.2 | 96.8 | 97.2 | 97.6 | 99.6 | 100 |
|  | (185) | (20) | (15) | **(6)** | (4) | (3 | (2) | (3) | (4) | (1) | (1) | (5) | (1) |
| *Enterobacter cloacae* (500) |  |  |  |  |  |  |  |  |  |  |  |  |  |
| Gepotidacin |  |  |  |  |  | 0.4 | 5.8 | 47.0 | 73.4 | 86.6 | **93.2** | 98.2 | 100 |
|  |  |  |  |  |  | (2) | (27) | (206) | (132) | (66) | **(33)** | (25) | (9) |
| Ciprofloxacin | 63.4 | 67.0 | 70.8 | 74.8 | 79.2 | 83.4 | 86.0 | 88.2 | **90.6** | 92.0 | 94.8 | 96.4 | 100 |
|  | (317) | (18) | (19) | (20) | (22) | (21) | (13) | (11) | **(12)** | (7) | (14) | (8) | (18) |
| *Escherichia coli* (1,000) |  |  |  |  |  |  |  |  |  |  |  |  |  |
| Gepotidacin |  |  | 0.2 | 0.6 | 3.6 | 17.5 | 75.0 | **95.3** | 97.7 | 99.8 | 99.9 | 100 |  |
|  |  |  | (2) | (4) | (30) | (139) | (575) | **(203)** | (24) | (21) | (1) | (1) |  |
| Ciprofloxacin | 53.2 | 54.4 | 56.9 | 64.8 | 67.6 | 68.8 | 69.1 | 69.2 | 70.8 | 73.9 | 85.8 | **91.6** | 100 |
|  | (532) | (12) | (25) | (79) | (28) | (12) | (3) | (1) | (16) | (31) | (119) | **(58)** | (84) |
| *Klebsiella aerogenes* (250) |  |  |  |  |  |  |  |  |  |  |  |  |  |
| Gepotidacin |  | 0.4 | 0.8 | 0.8 | 1.2 | 4.4 | 42.4 | 88.8 | **94.8** | 97.6 | 99.6 | 100 |  |
|  |  | (1) | (1) | (0) | (1) | (8) | (95) | (116) | **(15)** | (7) | (5) | (1) |  |
| Ciprofloxacin | 82.8 | 86.4 | **90.0** | 91.2 | 91.6 | 95.6 | 96.4 | 97.6 | 98.4 | 98.8 | 99.6 | 100 |  |
|  | (207) | (9) | **(9)** | (3) | (1) | (10) | (2) | (3) | (2) | (1) | (2) | (1) |  |
| *Klebsiella oxytoca* (250) |  |  |  |  |  |  |  |  |  |  |  |  |  |
| Gepotidacin |  |  |  |  | 0.4 | 5.2 | 48.4 | **91.6** | 97.2 | 99.2 | 99.6 | 100 |  |
|  |  |  |  |  | (1) | (12) | (108) | **(108)** | (14) | (5) | (1) | (1) |  |
| Ciprofloxacin | 78.4 | 82.4 | 85.6 | 87.2 | 89.2 | **93.2** | 96.4 | 98.0 | 99.2 | 100 |  |  |  |
|  | (196) | (10) | (8) | (4) | (5) | **(10)** | (8) | (4) | (3) | (2) |  |  |  |
| *Klebsiella pneumoniae* (500) |  |  |  |  |  |  |  |  |  |  |  |  |  |
| Gepotidacin |  |  |  |  | 0.4 | 1.4 | 4.6 | 40.2 | 75.2 | 88.6 | **96.6** | 98.4 | 100 |
|  |  |  |  |  | (2) | (5) | (16) | (178) | (175) | (67) | **(40)** | (9) | (8) |
| Ciprofloxacin | 59.4 | 63.8 | 66.4 | 67.6 | 74.0 | 75.8 | 79.8 | 82.0 | 83.2 | 85.6 | 89.4 | **91.6** | 100 |
|  | (297) | (22) | (13) | (6) | (32) | (9) | (20) | (11) | (6) | (12) | (19) | **(11)** | (42) |
| *Proteus mirabilis* (250) |  |  |  |  |  |  |  |  |  |  |  |  |  |
| Gepotidacin |  |  |  | 0.4 | 1.2 | 2.8 | 7.6 | 22.8 | 63.6 | **91.2** | 96.4 | 98.8 | 100 |
|  |  |  |  | (1) | (2) | (4) | (12) | (38) | (102) | **(69)** | (13) | (6) | (3) |
| Ciprofloxacin | 58.0 | 62.8 | 64.0 | 66.4 | 68.0 | 71.2 | 76.0 | 78.8 | 82.8 | 87.6 | **94.0** | 94.0 | 100 |
|  | (145) | (12) | (3) | (6) | (4) | (8) | (12) | (7) | (10) | (12) | **(16)** | (0) | (15) |
| *Providencia rettgeri* (250) |  |  |  |  |  |  |  |  |  |  |  |  |  |
| Gepotidacin |  |  |  |  | 0.8 | 2.8 | 9.6 | 49.2 | 80.4 | **93.2** | 95.2 | 96.8 | 100 |
|  |  |  |  |  | (2) | (5) | (17) | (99) | (78) | **(32)** | (5) | (4) | (8) |
| Ciprofloxacin | 56.4 | 68.8 | 73.6 | 80.8 | 84.8 | 86.8 | 89.2 | **92.8** | 94.4 | 96.4 | 97.6 | 98.0 | 100 |
|  | (141) | (31) | (12) | (18) | (10) | (5) | (6) | **(9)** | (4) | (5) | (3) | (1) | (5) |
| *Enterococcus faecalis* (500) |  |  |  |  |  |  |  |  |  |  |  |  |  |
| Gepotidacin |  |  |  | 0.4 | 5.2 | 22.2 | 84.4 | **99.2** | 99.6 | 99.8 | 99.8 | 100 |  |
|  |  |  |  | (2) | (24) | (85) | (311) | **(74)** | (2) | (1) | (0) | (1) |  |
| Ciprofloxacin | 0.2 | 0.2 | 0.4 | 2.0 | 24.4 | 70.2 | 73.4 | 73.4 | 73.6 | 75.8 | 83.0 | **93.4** | 100 |
|  | (1) | (0) | (1) | (8) | (112) | (229) | (16) | (0) | (1) | (11) | (36) | **(52)** | (33) |
| *Staphylococcus saprophyticus* (250) |  |  |  |  |  |  |  |  |  |  |  |  |  |
| Gepotidacin |  | 30.0 | **91.6** | 95.2 | 98.0 | 99.2 | 100 |  |  |  |  |  |  |
|  |  | (75) | **(154)** | (9) | (7) | (3) | (2) |  |  |  |  |  |  |
| Ciprofloxacin |  |  |  | 66.0 | **99.2** | 100 |  |  |  |  |  |  |  |
|  |  |  |  | (165) | **(83)** | (2) |  |  |  |  |  |  |  |

^a^ MIC_90_ is in boldface for each MIC distribution.

**TABLE S2** MIC values, MBC values, and MBC/MIC ratios for gepotidacin against 50 isolates

| **Organism** | **Reference Number** | **Gepotidacin MIC (µg/mL)** | **Gepotidacin MBC (µg/mL)** | **MBC/MIC ratio** |
| --- | --- | --- | --- | --- |
| *Citrobacter koseri* | 1985132 | 1 | 1 | 1 |
| *Citrobacter koseri* | 2022287 | 1 | 1 | 1 |
| *Citrobacter koseri* | 1991608 | 2 | 2 | 1 |
| *Citrobacter koseri* | 2022204 | 2 | 2 | 1 |
| *Citrobacter koseri* | 2022281 | 1 | 2 | 2 |
| *Enterobacter cloacae* | 2163424 | 8 | 8 | 1 |
| *Enterobacter cloacae* | 2044594 | 32 | 32 | 1 |
| *Enterobacter cloacae* | 1966259 | 4 | 8 | 2 |
| *Enterobacter cloacae* | 1999795 | 4 | 8 | 2 |
| *Enterobacter cloacae* | 2069808 | 4 | >128 | ≥64 |
| *Escherichia coli* | 1992367 | 1 | 1 | 1 |
| *Escherichia coli* | 2218901 | 2 | 2 | 1 |
| *Escherichia coli* | 1986037 | 1 | 2 | 2 |
| *Escherichia coli* | 2218900 | 4 | 8 | 2 |
| *Escherichia coli* | 1991025 | 4 | 32 | 8 |
| *Klebsiella aerogenes* | 2022262 | 1 | 1 | 1 |
| *Klebsiella aerogenes* | 1972851 | 4 | 4 | 1 |
| *Klebsiella aerogenes* | 1990204 | 4 | 4 | 1 |
| *Klebsiella aerogenes* | 2162922 | 4 | 4 | 1 |
| *Klebsiella aerogenes* | 2063272 | 4 | 16 | 4 |
| *Klebsiella oxytoca* | 2012978 | 2 | 2 | 1 |
| *Klebsiella oxytoca* | 2064317 | 2 | 2 | 1 |
| *Klebsiella oxytoca* | 2071244 | 2 | 2 | 1 |
| *Klebsiella oxytoca* | 2042240 | 1 | 2 | 2 |
| *Klebsiella oxytoca* | 2082943 | 4 | >128 | ≥64 |
| *Klebsiella pneumoniae* | 2171464 | 4 | 4 | 1 |
| *Klebsiella pneumoniae* | 1985547 | 4 | 8 | 2 |
| *Klebsiella pneumoniae* | 1991619 | 4 | 8 | 2 |
| *Klebsiella pneumoniae* | 2266717 | 4 | 8 | 2 |
| *Klebsiella pneumoniae* | 2266859 | 4 | 8 | 2 |
| *Proteus mirabilis* | 1990170 | 8 | 8 | 1 |
| *Proteus mirabilis* | 2092494 | 8 | 8 | 1 |
| *Proteus mirabilis* | 2267743 | 8 | 8 | 1 |
| *Proteus mirabilis* | 1992274 | 16 | 16 | 1 |
| *Proteus mirabilis* | 2162905 | 16 | 16 | 1 |
| *Providencia rettgeri* | 1965734 | 2 | 2 | 1 |
| *Providencia rettgeri* | 1976807 | 2 | 2 | 1 |
| *Providencia rettgeri* | 1873290 | 4 | 4 | 1 |
| *Providencia rettgeri* | 2082917 | 8 | 8 | 1 |
| *Providencia rettgeri* | 1922492 | 16 | 16 | 1 |
| *Enterococcus faecalis* | 1540655 | 1 | 1 | 1 |
| *Enterococcus faecalis* | 1810339 | 2 | 2 | 1 |
| *Enterococcus faecalis* | 1974556 | 4 | 4 | 1 |
| *Enterococcus faecalis* | 1984217 | 1 | 2 | 2 |
| *Enterococcus faecalis* | 1563710 | 2 | 4 | 2 |
| *Staphylococcus saprophyticus* | 1071860 | 0.06 | 0.06 | 1 |
| *Staphylococcus saprophyticus* | 979121 | 0.12 | 0.12 | 1 |
| *Staphylococcus saprophyticus* | 1428867 | 0.12 | 0.12 | 1 |
| *Staphylococcus saprophyticus* | 979122 | 0.06 | 0.12 | 2 |
| *Staphylococcus saprophyticus* | 1431962 | 0.06 | 0.12 | 2 |

**TABLE S3** Distribution of isolates tested by bacterial species and global region

| Bacterial species | Global region  No. of isolates | | | | | | |  |
| --- | --- | --- | --- | --- | --- | --- | --- | --- |
|  | Africa | Asia | Europe | Latin  America | Middle  East | North  America | South  Pacific | No. of isolates |
| *Citrobacter amalonaticus* |  |  |  |  |  | 7 |  | 7 |
| *Citrobacter braakii* |  |  | 3 |  |  | 4 |  | 7 |
| *Citrobacter farmeri* |  |  |  | 1 |  | 1 |  | 2 |
| *Citrobacter freundii* | 2 | 8 | 25 | 4 | 1 | 85 | 1 | 126 |
| *Citrobacter koseri* | 1 | 13 | 21 | 8 | 4 | 56 | 3 | 106 |
| *Citrobacter murliniae* |  |  | 1 |  |  |  |  | 1 |
| *Citrobacter youngae* |  |  |  |  |  | 1 |  | 1 |
| *Enterobacter cloacae* | 12 | 25 | 100 | 42 | 7 | 308 | 6 | 500 |
| *Enterococcus faecalis* | 12 | 29 | 100 | 26 | 20 | 306 | 7 | 500 |
| *Escherichia coli* | 32 | 67 | 200 | 56 | 21 | 612 | 12 | 1,000 |
| *Klebsiella aerogenes* | 2 | 16 | 50 | 21 | 2 | 153 | 6 | 250 |
| *Klebsiella oxytoca* | 4 | 16 | 50 | 10 | 6 | 156 | 8 | 250 |
| *Klebsiella pneumoniae* | 9 | 29 | 100 | 34 | 17 | 305 | 6 | 500 |
| *Proteus mirabilis* | 10 | 15 | 50 | 16 | 2 | 152 | 5 | 250 |
| *Providencia rettgeri* | 2 | 5 | 50 | 12 | 3 | 164 | 14 | 250 |
| *Staphylococcus saprophyticus* | 1 | 8 | 142 | 21 | 3 | 58 | 17 | 250 |
| No. of isolates | 87 | 231 | 892 | 251 | 86 | 2,368 | 85 | 4,000 |
| Percentage of total no. of isolates | 2.2 | 5.8 | 22.3 | 6.3 | 2.2 | 59.2 | 2.1 | 100 |

##

**TABLE S4** Distribution of isolates tested by bacterial species and year of collection

| Bacterial species | Year of collection  No. of isolates | | | | | | | | | Percentage of total no. of isolates |  |
| --- | --- | --- | --- | --- | --- | --- | --- | --- | --- | --- | --- |
|  | 2012 | 2013 | 2014 | 2015 | 2016 | 2017 | 2018 | 2019 | 2020 |  | No. of isolates |
| *Citrobacter amalonaticus* |  |  |  |  |  |  |  | 6 | 1 | 0.2 | 7 |
| *Citrobacter braakii* |  |  |  |  |  |  |  | 5 | 2 | 0.2 | 7 |
| *Citrobacter farmeri* |  |  |  |  |  |  |  | 2 |  | 0.1 | 2 |
| *Citrobacter freundii* |  |  |  |  |  |  |  | 105 | 21 | 3.2 | 126 |
| *Citrobacter koseri* |  |  |  |  |  |  |  | 89 | 17 | 2.7 | 106 |
| *Citrobacter murliniae* |  |  |  |  |  |  |  |  | 1 | <0.1 | 1 |
| *Citrobacter youngae* |  |  |  |  |  |  |  | 1 |  | <0.1 | 1 |
| *Enterobacter cloacae* |  |  |  |  |  | 48 | 112 | 265 | 75 | 12.5 | 500 |
| *Enterococcus faecalis* |  |  |  | 32 | 51 | 43 | 69 | 246 | 59 | 12.5 | 500 |
| *Escherichia coli* |  |  |  |  |  |  |  | 860 | 140 | 25.0 | 1,000 |
| *Klebsiella aerogenes* |  |  |  |  |  |  | 55 | 133 | 62 | 6.3 | 250 |
| *Klebsiella oxytoca* |  |  |  |  |  |  | 8 | 196 | 46 | 6.3 | 250 |
| *Klebsiella pneumoniae* |  |  |  |  |  |  |  | 396 | 104 | 12.5 | 500 |
| *Proteus mirabilis* |  |  |  |  |  |  |  | 213 | 37 | 6.3 | 250 |
| *Providencia rettgeri* |  |  |  |  | 2 | 11 | 36 | 177 | 24 | 6.3 | 250 |
| *Staphylococcus saprophyticus* | 4 | 4 | 33 | 24 | 24 | 39 |  | 122 |  | 6.3 | 250 |
| Percentage of total no. of isolates | 0.1 | 0.1 | 0.8 | 1.4 | 1.9 | 3.5 | 7.0 | 70.4 | 14.7 | 100 |  |
| No. of isolates | 4 | 4 | 33 | 56 | 77 | 141 | 280 | 2,816 | 589 |  | 4,000 |
